# News and Events of the Week

**Published:** 1910-12-03

**Authors:** 


### News and Events of the Week.

On Thursday, December 8, at 7 p.m., a meeting of the Royal Sanitary Institute will be held at 90 Buckingham Palace Road, when a paper will be read on "Camp Sanitation," by R. Basford, A.R. San. Inst. The chair will be taken at 7 p.m. by Louis C. Parkes, M.D., D.P.H. (Chairman of the Council of the Institute). General discussion is invited.

The Essex Education Committee reports that as a result of the medical inspection of new scholars entering secondary schools a proportion of nearly one-third in 700 were found to be in need of attention, principally to teeth and eyes. It has been decided to obtain a further report as to how far advice given to the parents was acted on.

London University—Presentation Day.—The presentation of degrees at the University of London was held on Monday last at South Kensington, having been postponed from May 6 on account of the national mourning. The day chosen, November 28, coincides with the anniversary of the foundation of the University, a date which it has been proposed shall in future always be commemorated as Founders' Day. Fears that the Chancellor, Lord Rosebery, might be prevented from attending by political calls proved unfounded, and the ceremony was robbed of none of its splendour. The number of graduates eligible for presentation ran into well over four figures, and it is certainly not a matter for regret that they did not all attend in person. As it was, the procession on Monday contained some 450 men and women, and the task of handing the parchments was no light one, as Lord Rosebery found. This part of the proceedings was enlivened, as usual, by undergraduate humour; but, fortunately, there was no attempt at the rowdyism now prevalent at some universities. Indeed, a further development of the rather crude forms of wit displayed would, perhaps, not be unacceptable to the audience patiently waiting during this somewhat tedious period. A novel and encouraging sight was the strong muster of members of the Officers' Training Corps, who also furnished the guard of honour for the Chancellor. Prof. M. J. M. Hill, Vice-Chancellor, who presented War Office Certificates, expressed the hope that before long work in the corps would be recognised as part of the qualification for a degree. The Chancellor's procession included many distinguished guests, senators, mayors and officials, among whom was the first holder of the newly-created office of Public Orator, Prof. Gardener. Only five medical schools were represented officially. Of the medical graduates only 15 out of 113 with the degree of M.B., B.S. appeared in person, but 16 of the 48 M.D.s did so. In his report as Principal, Dr. Miers referred to the urgent need of a new chemical laboratory for University College, and to the important new scheme of tutorial classes for the working people of London. Lord Rosebery, who was given a typical and cheery reception by the undergraduates, mentioned that the honorary degrees of the University were so select that only three existed, two of these being held by our Sovereign. He emphasised the needs of the University both financially and in regard to the grossly inadequate accommodation, but pointed out that the City companies gave a large bounty, and that grants were received from the London County Council, which, he thought, was proud to walk hand-in-hand with the University of London.

Prince Arthur of Connaught has consented to become president of the Appeal Committee for New Chemical Laboratories at University College, London, of which Sir Henry Roscoe is chairman. The object of the appeal is to raise £70,000 for the purchase of a site in Gower Place and the erection thereon of new chemical laboratories. The fund has now reached £10,156, and an effort is being made to raise at least £25,000, the cost of the site, before Christmas Day. Subscriptions or donations may be addressed to his Royal Highness at University College, and will be gratefully acknowledged by him, or to the Chairman, the Right Hon. Sir Henry Roscoe, at the same address.

The following are the arrangements at the Central London Throat and Ear Hospital, Gray's Inn Road, W.C., for the week. Lecture: Tuesday, December 6, at 3.45 p.m.: "External and Middle Ear," Mr. Chichele Nourse; Friday, December 9, 3.45 p.m.: "Clinical Cases," Dr. Abercrombie.

The following are the arrangements for next week at the Medical Graduates' College and Polyclinic, 22 Chenies Street, W.C.: Monday, December 5, at 4 p.m., Dr. S. E. Dore, Clinique (skin); at 5.15 p.m., Dr. W. W. H. Tate, "The Significance of Pain as a Symptom of Disease of the Pelvic Organs." Tuesday, December 6, at 4 p.m., Dr. James Collier, Clinique (medical); at 5.15 p.m., Dr. Harry Campbell, "The Treatment of Bronchitis in the Aged and Feeble." Wednesday, December 7, at 4 p.m., Mr. B. Mower White, Clinique (surgical); at 5.15 p.m., Dr. D'Oyly Grange, "Infective Arthritis." Thursday, December 8, at 4 p.m., Dr. Russell Wells, Clinique (medical); at 5.15 p.m., Mr. Somerville Hastings, "The Results of Nasal Obstruction." Friday, December 9, at 4 p.m., Dr. George Thompson, Clinique (eye).

The following are the arrangements for next week at the West London Post-graduate College: December 5 and 8, at 10 a.m., demonstration by Surgical Registrar; December 9, at 10 a.m., demonstration by Medical Registrar; December 6, at 11.30 a.m., demonstration of minor operations; December 5, at 12 noon, pathological demonstration; December 7, at 12.15 p.m., lecture, Dr. Grainger Stewart, "Practical Medicine." Lectures at 5 p.m.: December 5, Mr. Bidwell, "Surgical Treatment of Hæmorrhage from Gastric and Duodenal Ulcer"; December 6, Dr. Seymour Taylor, "Pneumonia"; December 7, Dr. Beddard, "Practical Medicine"; December 8, Dr. Bernstein, "Clinical Pathology"; December 9, Mr. Baldwin, "Practical Surgery."

According to the "London Gazette" his Majesty the King has been graciously pleased to award the Edward Medal of the Second Class to Mr. Edwin Arthur Dando, M.R.C.S., L.R.C.P., for courage shown on the occasion of the underground fire which occurred at the Russell Colliery, near Dudley, on April 17, 1910, and in connection with which the Edward Medal was awarded on July 23, 1910, to Arthur Cartwright, Isaiah Walker, Samuel Slater, and Anthony Willetts. It has since been brought to his Majesty's notice that Dr. Dando, who was summoned to the mine when the fire broke out, went down the pit and bravely assisted in the rescue work for several hours. He was at last overcome by the poisonous gases, and was brought to the surface unconscious, and he has not yet completely recovered from the effects of his courageous action.

